# Social Isolation During COVID-19 Pandemic: Challenges of a Rhinophyma Patient Reluctant to Seek Treatment

**DOI:** 10.7759/cureus.82207

**Published:** 2025-04-13

**Authors:** Chelsea Yim, Jeewon Chon, Peter Laub, Joseph Ogrodnik

**Affiliations:** 1 Department of Plastic and Reconstructive Surgery, Loyola University Medical Center, Chicago, USA

**Keywords:** case report, covid-19, patient education, rhinophyma, social isolation

## Abstract

Rhinophyma is a progressive dermatologic condition of the nose that is characterized by hypertrophic thickening of the skin, edema of the nasal pyramid, and hyperplasia of nasal sebaceous glands and connective tissue. We present a case of severe rhinophyma, where the patient delayed seeking treatment due to social isolation during COVID-19 and apprehension about surgery. Mask-wearing concealed the deformity and allowed it to worsen. We will demonstrate the surgical technique with a photo timeline, illustrating healing progression. This is a useful adjunct when counseling patients with rhinophyma on the expected post-operative course and appearance. A 67-year-old male patient presents with enlarging cysts and thickening epidermis on the nose resulting in large hypertrophic, nodular lesions and telangiectasias. His nose was deformed with multiple pedunculated lesions from the nasal dorsum, sidewalls, and along the alar rims. The largest lesion measured 5 cm; six were excised using electrocautery. Each sebaceous cyst was excised individually to create a more uniform surface. The remaining skin had severe rhinophyma. Loop electrocautery (LP) was used to excise the nasal dorsum and bilateral nasal sidewalls, the alar rims over the soft tissue triangle, and the nasal tip and over the columella. A 5-mm tip was used for finer elements, producing an improved nasal shape, and no cartilage was exposed. The nose was dressed with antibiotic ointment and nonadherent gauze with absorptive gauze overlay. Several serial photos will demonstrate rapid healing. The patient’s nose was fully epithelized one month post-operatively with much-improved contour and shape. Primary excision of nodular cystic lesions followed by LP can help restore normal nasal appearance in patients with deforming rhinophyma. Healing via secondary intention with daily dressing changes remains an excellent strategy for patients' status post rhinophyma excision. The photo timeline of this severe rhinophyma case is a valuable tool when counseling future patients who are apprehensive about undergoing surgical intervention.

## Introduction

Rhinophyma is a progressive, dermatological condition of the nose that is characterized by hypertrophic thickening of the skin, edema of the nasal pyramid, and hyperplasia of nasal sebaceous glands and connective tissue. Although it has a well-documented association with rosacea, the etiology of rhinophyma development is hypothesized to be multifactorial [1-3]. Nevertheless, rhinophyma may cause severe psychosocial distress for patients and anatomical defects that can cause nasal obstruction. Here, we describe a case of severe rhinophyma, in which the patient delayed seeking treatment due to his social isolation during the COVID-19 pandemic and his apprehension about surgery.

## Case presentation

**Figure 1:**
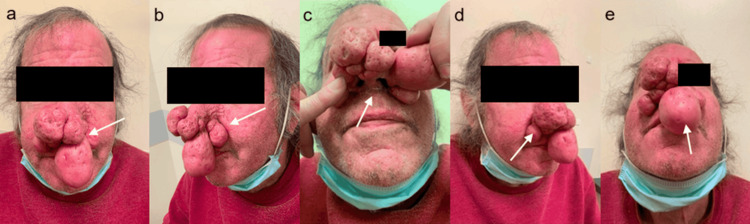
(a-e) Preoperative View of Rhinophyma at Consultation

A 67-year-old Caucasian male patient presents with a history of enlarging masses and thickening epidermis on the nose resulting in large hypertrophic, nodular lesions and multiple telangiectasias (Figure 1). Social isolation during COVID-19 and lack of patient education made him reluctant to seek medical care. Mask-wearing also concealed the deformity, allowing the lesion to grow large enough to obstruct the nostrils and make personal grooming difficult.

After presenting with physical symptoms, the patient finally sought care after his friend encouraged him to see a physician. He originally avoided any medical treatment due to the belief that his only solution was a rhinectomy, and this avoidance was exacerbated by social isolation during the pandemic. However, after extensive counseling about rhinophyma excision, the patient agreed to the procedure. His main concerns were regarding recovery and wound healing. A photo timeline would have been useful in these counseling sessions to further educate and comfort the patient.

The patient underwent rhinophyma excision surgery to improve his quality of life. He had a deformed external nose with multiple pedunculated lesions from the nasal dorsum and the sidewalls and along the alar rims. The largest and smallest lesions measured 5 and 1.5 cm, respectively. Given the extent of his disease, our team chose to proceed with electrocautery and performed the case under general anesthesia. Local anesthesia with epinephrine was used to reduce pain and reduce blood loss. Six lesions were excised using this technique. First, each mass was excised individually at the base to create a more uniform surface. Next, 10- and 5-mm diameter loop electrocautery tips were utilized to sculpt the nose, leaving a thick layer of dermis.

**Figure 2:**
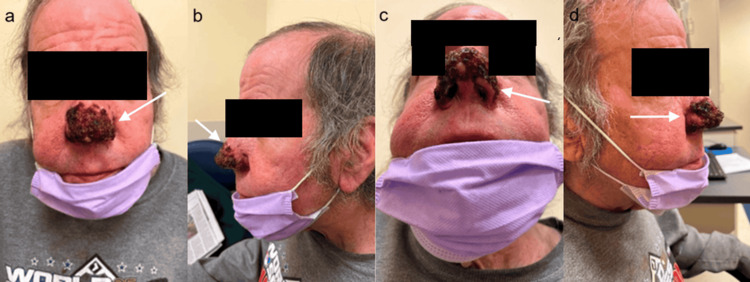
(a-d) Post-operative View of Rhinophyma Left to Heal via Secondary Intention 11 Days Post-op

**Figure 3:**
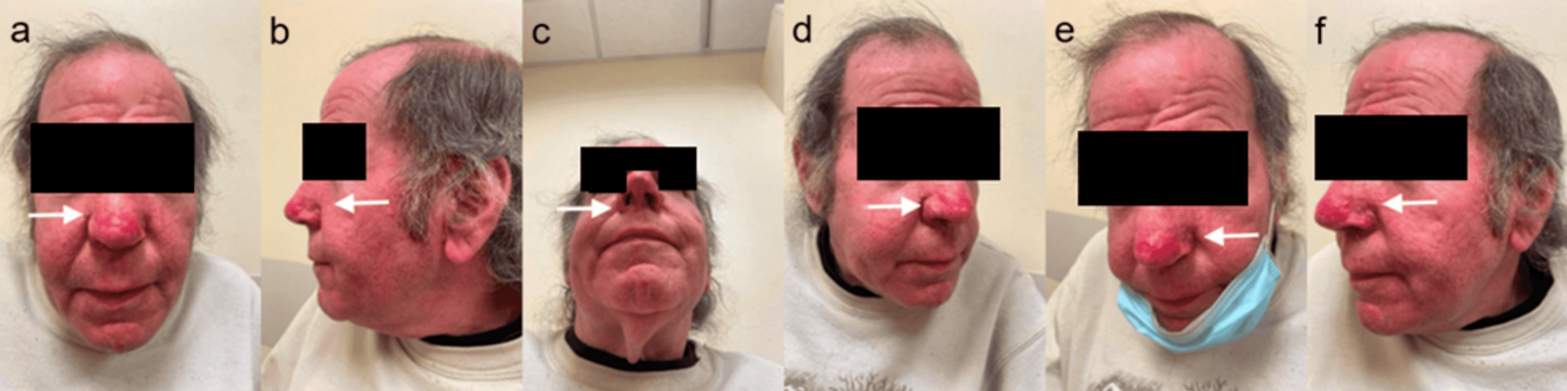
(a-f) Post-operative View of Rhinophyma 12 Weeks Post-op

**Figure 4:**
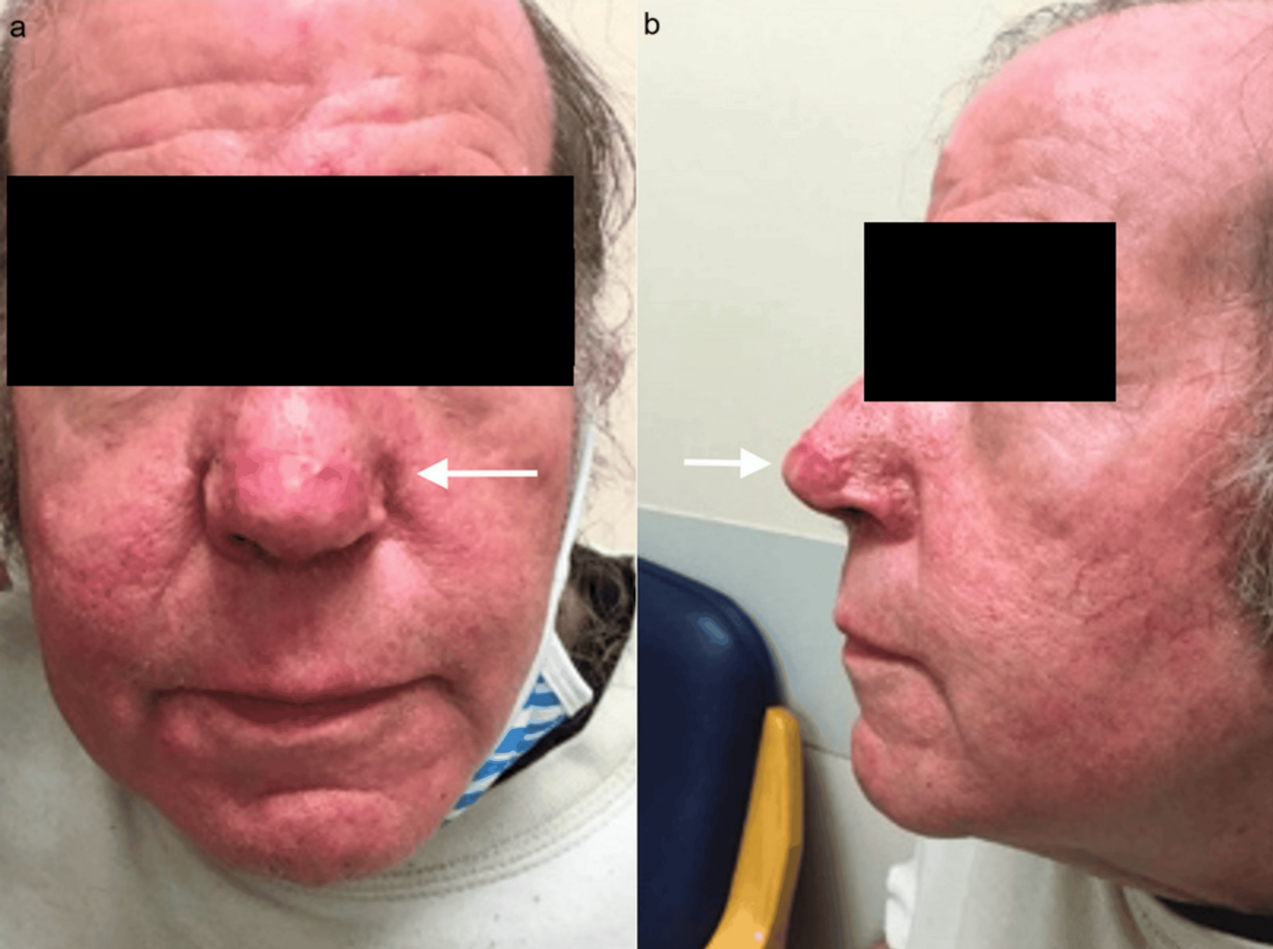
(a, b) Post-operative View of Rhinophyma 21 Weeks Post-op

**Figure 5:**
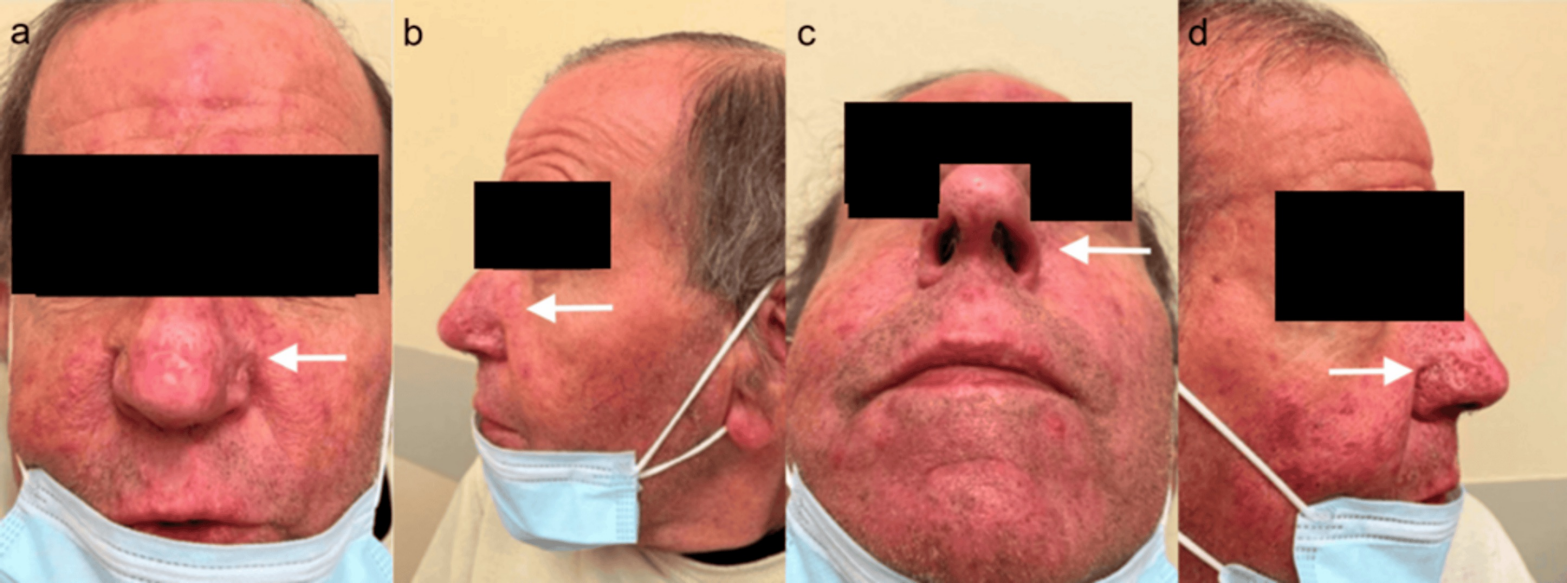
(a-d) Post-operative View of Rhinophyma 34 Weeks Post-op

**Figure 6:**
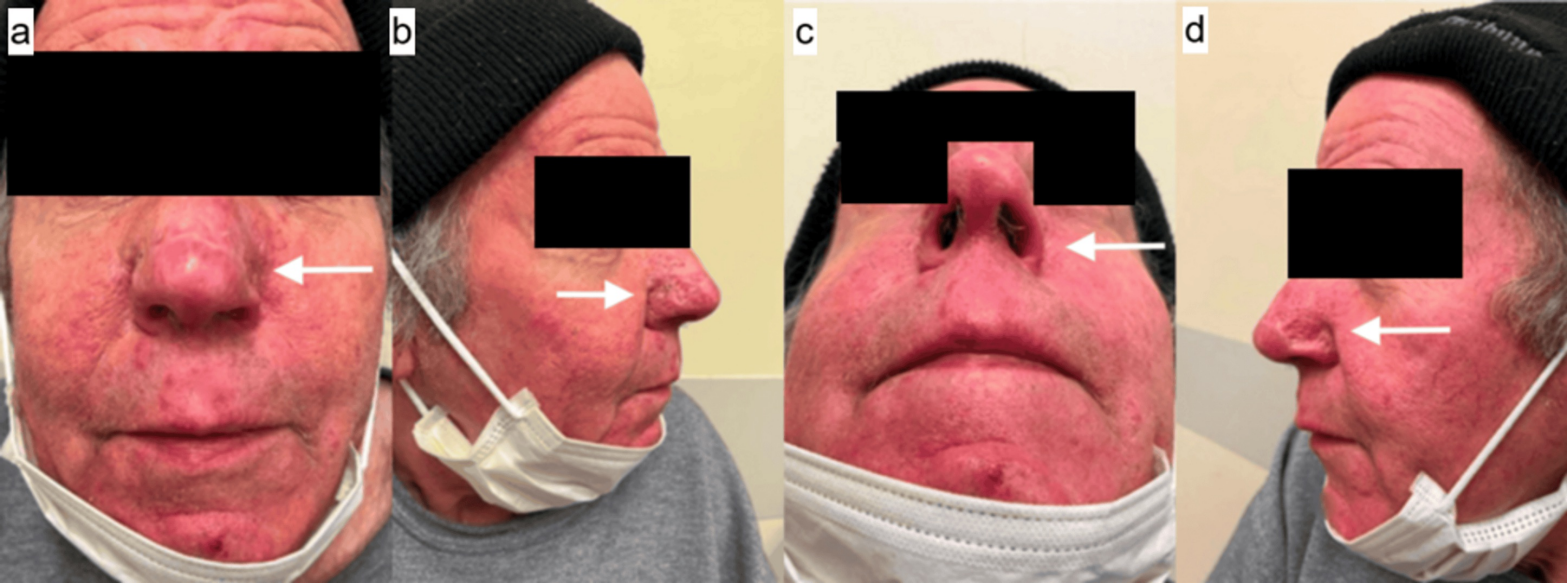
(a-d) Post-operative View of Rhinophyma 46 Weeks Post-op

A 5-mm tip was used for finer elements such as the alar groove and soft triangle, producing an improved nasal shape. Care was taken to ensure cartilage was not exposed, and electrocautery was used to obtain hemostasis. There was an immediate improvement in the opening of the nares. The rhinophyma excision site was left to heal by secondary intention and was dressed in bacitracin/generic petroleum gauze and silver-impregnated absorptive gauze. After the dressing was removed on post-op day five, the wound was managed daily with soap and water washes then bacitracin. The patient’s nose was fully epithelized one month post-operatively with markedly improved contour and shape. The patient continued to show excellent results post-operatively (Figures 2-6).

## Discussion

Rhinophyma is defined as a disfiguring, abnormally shaped benign bulbous growth on the nose due to hyperplasia of nasal sebaceous glands and connective tissue [1]. It characteristically contains background telangiectasias and thickened hyperplastic skin and was first linked to late-stage rosacea in 1846 by Ferdinando Hebra Von (1816-1880) [2]. However, the etiology of rhinophyma development is still hypothesized to be multifactorial. It has been thought that increased activation of the immune system coupled with dysregulation of inflammatory cells, vasculature, and lymphatic vessels is responsible for the development of rhinophyma. Increased activation of the immune system, mast cell proliferation, and vasodilation encourage angiogenesis through increased levels of vascular endothelial growth factor and fibroblast growth factor, which result in increased vascular permeability, edema, and erythema [3-5]. Further histamine dysregulation results in persistent inflammation, leading to hyperplasia of the sebaceous glands [6]. Other hypothesized triggers for rhinophyma development include microorganisms such as *Demodex folliculorum* and *Helicobacter pylori*, trauma, sun, or ultraviolet (UV) light [3-5,7].

Treatment options for rhinophyma removal include excisional, ablative, and laser procedures. Prior to surgery, patients must not be taking isotretinoin as it negatively affects reepithelization [8,9].

Excisional methods include full-thickness resection with reconstruction via flap or grafts, partial-thickness tissue removal, methods such as cryosurgery, "heated” and “cold” scalpel excision, harmonic ultrasound scalpel, and dermabrasion. While most of these techniques have fallen out of favor, partial-thickness tissue removal and dermabrasion are still used today. Partial-thickness tissue removal excises superficial tissue and preserves the pilosebaceous unit. It is performed with a standard scalpel but can result in scarring and unintentional removal of the sebaceous unit [10]. Dermabrasion is a technique used in conjunction with other treatments for more precise contouring [11].

Ablative techniques include electrosurgery, electrocautery, coblation, or radiofrequency to reduce the appearance of rhinophyma. In electrocautery, there is minimal atrophic scarring with a smoothing effect. However, a main drawback of these methods is the increase in heat in local tissues, which can lead to necrosis if not monitored correctly. “Cold coblation” avoids this side effect by using radiofrequency to excite electrons to de-epithelize tissue at low temperatures to reduce temperature-induced damage [12]. However, several studies demonstrated that electrocautery and surgical excision are effective treatments for rhinophyma, providing excellent esthetic results with minimal risks of complications. Scalpel excision allows for precise shaping and contouring, while electrosurgery efficiently removes excess tissue with minimal bleeding. Several cases also reported no hyperpigmentation or scarring, further supporting the efficacy of these techniques in treating rhinophyma [7,13-15].

Directed laser energy techniques include carbon dioxide lasers, diode lasers, and erbium:yttrium aluminum garnet (YAG) in combination with carbon dioxide lasers. Carbon dioxide lasers yield positive results but can be associated with pain with local anesthetic, hyperpigmentation, scarring, and open pores [16,17].

While there has been no consensus on the best form of treatment, excision with loop electrocautery is an older technique that is highly recommended for patients who are older and more prone to bleeding [18]. Moreover, wire loop electrocautery is a cost-effective and simple technique to excise rhinophyma [19].

**Figure 7:**
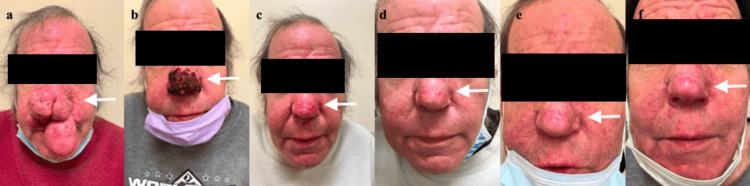
Complete Rhinophyma Excision Photo Timeline, Frontal view. (a) Consultation; (b) 11 Days Post-op; (c) 12 Weeks Post-op; (d) 21 Weeks Post-op; (e) 34 Weeks Post-op; (f) 46 Weeks Post-op

**Figure 8:**
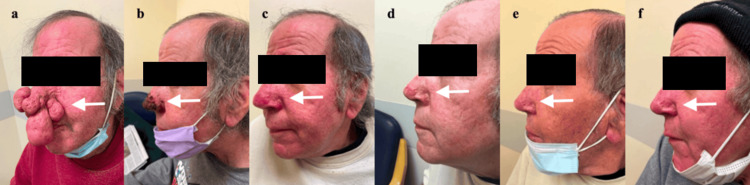
Complete Rhinophyma Excision Photo Timeline, Left Lateral View. (a) Consultation; (b) 11 Days Post-op; (c) 12 Weeks Post-op; (d) 21 Weeks Post-op; (e) 34 Weeks Post-op; (f) 46 Weeks Post-op

We demonstrated the electrocautery surgical technique with a photo timeline showing the healing progression (Figures 7, 8). This photo timeline is a useful adjunct when counseling patients with rhinophyma on the expected post-operative course and appearance to encourage medical intervention for rhinophyma. This has illustrated that primary excision of the nodular cystic lesion followed by looped electrocautery can be used to help restore normal nasal appearance in patients with deforming rhinophyma. Healing via secondary intention with daily dressing changes remains an excellent strategy for patients’ status post rhinophyma excision, as seen with this patient’s photo results on day five and one month post-op. Most importantly, the patient was very satisfied with the results and desires no further interventions to improve appearance.

In cases where patients have underlying rosacea, education on lifestyle modifications and adherence to medical therapy can prevent recurrence. Education about post-operative care, such as proper wound care and avoiding triggers like UV exposure, can enable proper compliance. Ultimately, well-informed patients are more likely to seek treatment early, achieve optimal outcomes, and have higher levels of satisfaction.

Patient education plays a pivotal role in rhinophyma management, especially as early detection of rhinophyma warning signs can massively aid in symptom management. It also aids in addressing misconceptions about the condition and empowering informed, swift decision-making. Many patients associate rhinophyma with irreversible disfigurement or believe surgery involves significant risks that may worsen disfigurement. Similarly, our patient was fearful of surgical treatment and delayed seeking medical treatment until his rhinophyma lesions became severely and unmanageably large. COVID-19 policies provided an opportunity to conceal his condition to avoid possible social ridicule and other psychosocial symptoms. Patients with apprehension and minimal to no education regarding surgical intervention are also likely to postpone their treatment, worsening their condition. Comprehensive counseling about disease progression, treatment efficacy, and recovery timelines is essential to dispel myths and promote timely intervention. Our report aims to provide a realistic photo timeline that will remove the guesswork that patients have about their procedure and set realistic expectations for their outcomes.

## Conclusions

This case report highlights the challenges faced by a patient with rhinophyma during the COVID-19 pandemic, which resulted in delayed medical treatment due to social isolation and apprehension about surgery. Rhinophyma is a progressive dermatological condition of the nose that can cause severe psychosocial distress and anatomical defects. Rhinophymas are treated primarily through surgical excision. The three main categories of surgical excision are mechanical destruction, directed electrical energy or radiofrequency, and directed laser energy. The surgical technique demonstrated in this case report, primary excision of the nodular cystic lesion followed by looped electrocautery, can help restore normal nasal appearance and function in patients with deforming rhinophyma. The photo timeline presented in this report is a useful adjunct when counseling patients with rhinophyma on the expected post-operative course and appearance to encourage medical intervention for rhinophyma. Overall, this report aims to provide reassurance for patients regarding surgery and encourage patients to seek medical treatment for rhinophyma.
